# Qualification of Dentists in Prussia

**Published:** 1836-04

**Authors:** 


### QUALIFICATION OF DENTISTS IN PRUSSIA.

By a government order, dated the 29th April, 1835, directed to the different governments and medical colleges of the kingdom, it is decreed, that no person shall be admitted to examination for a licence to practise as a dentist who, in additlon to his testimonials of having acquired practical dexterity in his art with a licensed and practising dentist, shall not adduce proofs of being in one or other of the following conditions:

1. Of being a licensed physician or surgeon;
2. Of having served three years as surgeon in the army;
3. Of having obtained such knowledge and skill as are necessary for a surgeon in a regular attendance at places of public education.

In reference to this last condition, he must bring testimonials of having attended, during a curriculum of two years, lectures on anatomy; the theory of medicine; general, special, and operative surgery; a chirurgical clinic, and, when practicable, particularly on dental surgery.

*Rust's Mag.* B. 45, Heft 1, 1835.

---

ENCOURAGEMENT OF VACCINATION IN PRUSSIA.

